# Storage stability of lysostaphin solution and its pulmonary delivery

**DOI:** 10.1007/s13346-024-01518-9

**Published:** 2024-01-17

**Authors:** Ping Zeng, Pengfei Zhang, Ho Wan Chan, Shing Fung Chow, Jenny Ka Wing Lam, Margaret Ip, Sharon Shui Yee Leung

**Affiliations:** 1 School of Pharmacy, Faculty of Medicine, The Chinese University of Hong Kong, Hong Kong SAR, China; 2 Department of Pharmacology and Pharmacy, Li Ka Shing Faculty of Medicine, The University of Hong Kong, Hong Kong SAR, China; 3 Department of Pharmaceutics, UCL School of Pharmacy, University College London, 29-39 Brunswick Square, London, WC1N 1AX UK; 4 Department of Microbiology, Faculty of Medicine, The Chinese University of Hong Kong, Hong Kong SAR, China

**Keywords:** Inhalation therapy, Bacterial lung infections, Multidrug-resistant bacteria, MRSA, Bacteriolytic enzyme, Peptidoglycan

## Abstract

Methicillin-resistant *Staphylococcus aureus* (MRSA) has become a leading causative pathogen of nosocomial pneumonia with an alarming in-hospital mortality rate of 30%. Last resort antibiotic, vancomycin, has been increasingly used to treat MRSA infections, but the rapid emergence of vancomycin-resistant strains urges the development of alternative treatment strategies against MRSA-associated pneumonia. The bacteriolytic enzyme, lysostaphin, targeting the cell wall peptidoglycan of *S. aureus*, has been considered as a promising alternative for MRSA infections. Its proteinaceous nature is likely benefit from direct delivery to the lungs, but the challenges for successful pulmonary delivery of lysostaphin lying on a suitable inhalation device and a formulation with sufficient storage stability. In this study, the applicability of a vibrating mesh nebulizer (Aerogen Solo^®^) and a soft mist inhaler (Respimat^®^) was investigated. Both devices were capable of aerosolizing lysostaphin solution into inhalable droplets and caused minimum antibacterial activity loss. In addition, lysostaphin stabilized with phosphate-buffered saline and 0.1% Tween 80 was proved to have acceptable stability for at least 12 months when stored at 4 °C. These promising data encourage further clinical development of lysostaphin for management of MRSA-associated lung infections.

**Graphical abstract:**

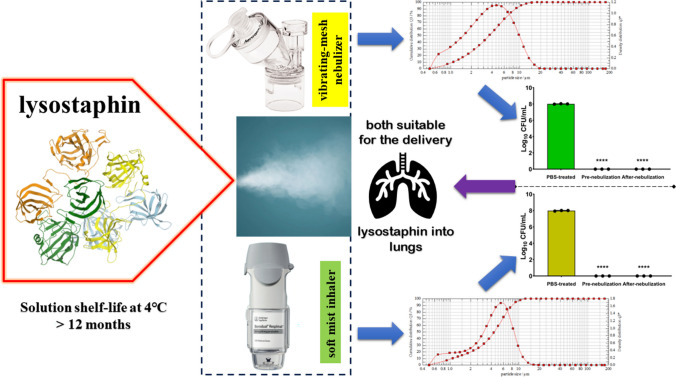

- Lysostaphin had insignificant activity loss after aerosol generation by a vibrating mesh nebulizer and a soft mist inhaler.
- Most of the lysostaphin aerosols generated by the vibrating mesh nebulizer and soft mist inhaler are inhalable.
- The vibrating mesh nebulizer and soft mist inhaler are suitable device for pulmonary delivery of lysostaphin.

## Introduction

Lower respiratory tract infections have been ranked as one of the top ten leading causes of death worldwide over the past two decades by the World Health Organization [1]. The increasing prevalence of antimicrobial resistance in the respiratory superbugs is one of the major contributors for the high mortality rates [2]. Among those, methicillin-resistant *Staphylococcus aureus* accounted for 20–40% of all hospital-acquired and ventilator-associated pneumonias [3], having an in-hospital mortality rate of ~ 30% [4, 5]. Although uncommon (0.51 to 0.64 cases per 100,000), MRSA also has been recognized as a cause of severe community-acquired pneumonia, which often occurs following influenza infection [6, 7], with a mortality rate as high as 56 to 63% [8–10]. MRSA are strains of *S. aureus* that are concomitantly resistant to most commonly used antibiotics, including methicillin, oxacillin, penicillin, amoxicillin, and cephalosporins, posing significant therapeutic challenges in controlling the related infections. Last resort antibiotic, vancomycin, has been increasingly used to treat MRSA infections, but outbreaks of vancomycin-resistant strains have been occasionally reported since 2002 [11, 12]. Therefore, urgent attention to discover novel anti-staphylococcal agents with effective delivery approaches to manage MRSA-associated pneumonia are required.

Lysostaphin is a well-known antibacterial enzyme derived from *Staphylococcus simulans* that specifically kills *S. aureus* by breaking the pentaglycine cross-links in the cell wall peptidoglycan [13]. The excellent bactericidal efficacy of lysostaphin had been demonstrated in vitro and in vivo, showing superiority to vancomycin and other antibiotic treatments [14, 15]. Therefore, there has been longstanding interest in developing lysostaphin as an alternative antibiotic for MRSA infections [16]. In 2010, Biosynexus Corp. developed a lysostaphin cream for *S. aureus* nasal decolonization and phase I/II clinical trials data established both safety and effectiveness, but its further development was halted because the clinical standard, mupirocin, came off patent protection [17]. With the rapid emergence of MRSA strains in recent years, lysostaphin re-appears to be a promising antibacterial candidate.

Being a protein from a bacterial source, there are concerns about enzyme degradation and immunogenicity of lysostaphin upon systemic delivery, leading to reduced antibacterial efficacy and poor safety profile in treating lung infections [18]. Therefore, directly delivering lysostaphin to the lungs would be a more rational approach in achieving optimal concentration at the target site, with minimum systemic exposure to minimized unwanted side effects. Liquid-based aerosol delivery of protein-based therapeutics using nebulizers have been the choice for their pulmonary delivery, ~ 75% of the developing inhaled protein therapeutics [19], as they do not require substantial formulation effort and can be delivered in higher dosages. Moreover, nebulizers require little patient coordination, allowing them to be easily used across a wide patient population, including children, the elderly and ventilated patients, particularly favorable in hospital settings. Recently, the capability of a hybrid surface and bulk acoustic wave platform (HYDRA) to nebulize lysostaphin was demonstrated [20], but this device is not commercially available yet. Respimat soft mist inhalers (SMIs), which have the advantages of portability and electricity-free operation, are another type of inhaler device have been demonstrated to be an efficient device to deliver solution-based medications to the lungs [20]. Its suitability in aerosolizing antibacterial enzymes has never been attempted before. This study first examined the feasibility of using the commercially available Aerogen Solo^®^ vibrating mesh nebulizer and Respimat^®^ Soft Mist Inhaler to deliver active lysostaphin into the lungs, likely used for nosocomial and outpatient pulmonary infection management, respectively. The storage stability of lysostaphin solution in a simple formulation system containing PBS and 0.1% Tween 80 was also evaluated.

## Materials and methods

### Materials

Lysostaphin (1200 U/mg) was purchased from Dieckmann (Shenzhen, China) and reconstituted in phosphate-buffered saline (PBS) with 0.1% Tween 80 to a concentration of 1 mg/ml. Nutrient broth (NB) and agar bacteriological (AGAR NO.1) were obtained from OXOID (Hampshire, UK). Mueller Hinton broth (MHB) was purchased from Hopebio (Qingdao, China). Dulbecco’s modified Eagle’s medium (DMEM), fetal bovine serum (FBS), and other chemicals were supplied by Sigma Aldrich (Saint Louis, MO, US) unless otherwise noted. Milli Q water with resistivity of 18.2 MΩ·cm at 25 °C was freshly dispensed from a Milli Q Integral water purification system (Burlington, MA, USA).

### Cell strains and culture conditions

Two clinical MRSA strains (MRSA-2 and MRSA-3) and human lung epithelial BEAS-2B cells were provided by the department of Microbiology at Prince of Wales Hospital, Hong Kong. Standard MRSA and methicillin-sensitive *S. aureus* (MSSA) strains acquired from the American Type Culture Collection (ATCC) were also tested. Table 1 summarized the characteristics of bacterial strains used in the present study. All the bacterial strains were grown in NB medium at 37 °C for overnight to obtain the stationary phase bacteria. Logarithmic-phase bacteria were obtained by diluting the overnight culture 100-fold in fresh NB for further incubation at 37 °C for 3–4 h until the optical density at 600 nm (OD_600_) reached ~ 0.6. BEAS-2B cells were incubated in a medium containing 90% DMEM and 10% FBS.

**Table 1:** MICs of methicillin, ampicillin, and lysostaphin against different *S. aureus* strains

**Antibiotics sensitivity**	**Bacterial strains**	**MIC (μg/ml)**		
		**Methicillin**	**Ampicillin**	**Lysostaphin**
Sensitive	ATCC 29213	1	< 0.5	4
Resistant	ATCC 1717	8	> 32	8
Resistant	ATCC 43300	2	4	8
Resistant	MRSA-2	4	< 0.5	1
Resistant	MRSA-3	> 32	32	8

### Minimum inhibition concentrations (MICs)

The MICs of lysostaphin and other commonly used antibiotics against various strains of *S. aureus* were determined according to the Clinical and Laboratory Standards Institute (CLSI) guidelines. Briefly, overnight bacterial culture was diluted with fresh MHB to a bacterial density of ~ 1 × 10^6^ CFU/ml. Lysostaphin or antibiotic solutions were added into individual wells of 96-well plates and performed 2-fold serial dilution, followed by the addition of the prepared bacterial inoculum. The plate was further incubated at 37 °C overnight. Wells with PBS and MHB were served as growth and background controls, respectively. The MICs were determined as the lowest concentration of lysostaphin/antibiotics that could inhibit the bacteria growth.

### Turbidity reduction assay (TRA)

TRA was then employed to confirm the bacteriolytic activity of lysostaphin. Briefly, 10 μL lysostaphin stock solution (1 mg/ml) was added to 190 μL PBS resuspended logarithmic-phase bacterial suspension of OD_600_ ~ 0.7 in 96-well plates. The OD_600_ values of the mixtures were measured at a specific time using a microplate reader (CLARIOstar, BMI Labtech, Germany). Wells with bacteria and PBS mixture were served as positive and negative controls, respectively.

### Cytotoxicity assay

BEAS-2B cells after reaching 90% confluency were incubated with lysostaphin for 24 h at 37 °C. Next, 20 μL of thiazolyl blue tetrazolium bromide (5 mg/ml in deionized water) was added for another 4-h staining. The mixed aqueous solution was then removed and a total of 150 μL of dimethylsulfoxide was added to dissolve sedimented formazan. After that, the OD_570_ values were measured by a microplate reader (CLARIOstar, BMI Labtech, Germany). Cell survival rate was calculated by % survival ratio = 1 − [(ABS_c_ − ABS_t_)/(ABS_c_ − ABS_b_) × 100%]. ABS_c_, ABS_t_, and ABS_b_ were defined as the OD_570_ values of the control groups (cells without any treatment), tested groups, and blank groups (DMSO only), respectively.

### Antibacterial activity in a co-culture system

BEAS-2B cells were infected with MRSA-2 at logarithmic phase for 1 h. Then, the co-culture system was treated with lysostaphin at varied concentrations for 4 h at 37 °C. After that, BEAS-2B cells were lysed with 0.1% EDTA. Homogeneous aliquots were diluted and dropped on the surface of MH agar plates for overnight incubation. Bacterial colonies (3–30) were counted and the primary colony formation unit (CFU) was calculated.

### Short-term environmental tolerance capability

MRSA-2 was selected as the model bacterial strain. Environmental tolerance capability under various pH (4–9), temperatures (20–70 °C), NaCl concentrations (150–600 mM), serum level (0–50% volume ratio), mannitol (1–10%), glycin (5–20 mM), and Tween 80 (0.1–0.5%) of lysostaphin was evaluated using TRA by recording the OD_600_ values after 2 h treatment at 37 °C. For the pH stability experiment, lysostaphin stock solution (1 mg/ml) was diluted into 100 mM citric acid-Na_2_HPO_4_ buffer (pH 4.0–8.0) or 100 mM glycin-NaOH buffer (pH 9.0) to a final concentration of 4 μg/ml. For the temperature stability experiment, lysostaphin diluted into PBS was incubated at 20, 37, 50, and 70 °C for 1 h before performing the TRA. To measure the antibacterial activity of lysostaphin in human serum, NaCl, mannitol, glycin, and Tween 80, logarithmic-phase bacteria were resuspended in PBS mixed with different concentration of to-be-tested substances for the TRA. Bacteria incubated with the PBS was served as the control in all experiments.

### Aerosolization of lysostaphin and activity loss

The clinically relevant Aerogen^®^ Solo vibrating mesh nebulizer with Pro-X Controller (Aerogen Ltd., Dangan, Galway, Ireland) and Respimat^®^ SMI (Boehringer Ingelheim Ltd., Ingelheim am Rhein, Germany) were used to aerosolize lysostaphin stock solution (1 mg/ml). For the vibrating mesh nebulizer, 500 μl lysostaphin stock solution was filled and nebulized for 1 min. For the SMI, 2 ml lysostaphin stock solution was filled into the cartridge and actuated 10 times. The weight difference before and after actuations were recorded to determine the ex-actuator dose per actuation. The aerosolized droplets were collected with a 50-ml Falcon tube sealed around the outlet of the inhaler devices, followed by centrifugation. The activity of aerosolized lysostaphin was determined by the antibacterial assay described above.

Aerosol particle size distribution of the aerosolized protein solutions (1 mg/ml) from an Aerogen^®^ solo nebulizer and a Respimat^®^ SMI was evaluated by laser diffraction using the HELOS Sympatec (GmbH, Clausthal-Zellerfeld, Germany). One-second measurements over 60 s of continuous nebulization were performed for the nebulizer measurement and measurement for 10 actuations were performed for the SMI. The results were expressed by the median diameter and the span which is defined as (D_90_-D_10_)/D_50_, where D_10_, D_50_, and D_90_ are the particle diameter at 10, 50, and 90 percentiles of the particle population, respectively. The data obtained were used to estimate regional deposition (lung and oropharynx) of the aerosols by the ARLA respiratory deposition calculator, https://sites.ualberta.ca/~arla/deposition_calculator.html [21]. The inhalation flow rate of 300 ml/s, tidal volume of 800 ml, and functional residual capacity of 3000 ml, which are comparable to typical adult male’s tidal breathing parameters, was used for the calculation.

### Circular dichroism (CD) spectroscopy

Far-UV CD spectroscopy (J-1500 CD spectrometer, Jasco Inc., Tokyo, Japan) was employed to evaluate changes in the secondary structure of lysostaphin upon the nebulization process. The spectrum measurement was performed with non-nebulized and nebulized lysostaphin samples diluted with deionized water to a final concentration of 10 μg/ml using a wavelength range from 190 to 260 nm at 25 °C. The spectra contribution from the cuvette and PBS/water mixture were subtracted from the sample spectra.

### Storage stability and shelf-life prediction

The activity of the PBS+ 0.1% Tween 80 reconstituted lysostaphin was evaluated immediately and after storage at 4, 20, 37, 50, and 60 °C using TRA and/or MIC assay. The degradation rate at each storage temperature was evaluated to estimate the shelf-life of lysostaphin solution. In brief, MRSA-2 cells at logarithmic phase (OD_600_ ~ 0.7) were treated with lysostaphin solutions (4 μg/ml) which were stored at different temperatures. After 2-h incubation, OD_600_ values of each group were recorded. MRSA-2 treated with freshly prepared lysostaphin solution and PBS was defined as 100% bioactivity and 0% bioactivity respectively. Collected data were fitted with first-order reaction kinetics (Eq. (1)) and the Arrhenius equation (Eq. (2)) to establish a shelf-life prediction model for lysostaphin solution.

1 $$\text{First-order:}\;k_dt=In(\frac{E_{\mathrm0}}E)$$

2 $$\text{Arrhenius equation:}-\!In(k_d)=\frac{E_a}{RT}-\mathrm{ln\;(A)}$$

where ${k}_{d}$ is the inactivation reaction rate constant; t = time (day); ${E}_{0}$ is the initial enzyme activity; ${E}_{0}$ is the residual enzyme activity at the sampling time; ${E}_{a}$ is the activation energy; R is the universal gas constant (8.314 J/mol/K); T is the thermodynamic temperature (K); and A is the frequency factor.

### Statistical analysis

All experiments were performed in three biological replicates and repeated at least two independent times unless specified. The aerosolization experiment and size measurement were triplicate. All results were expressed as mean ± one standard deviation. One-way analysis of variance (ANOVA) at a confidence level of 95% was employed to identify any statistically significant differences in cell and bacteria viability. A *p* value of < 0.05 was considered statistically significant.

## Results and discussion

### Antibacterial potential of lysostaphin

The MICs of lysostaphin against a panel of standard and clinical strains of *S. aureus*, both methicillin-sensitive and methicillin-resistant ones were first determined (Table 1). The MICs of lysostaphin against standard strains were found to be comparable to the reported values [22]. The susceptibility of bacteria towards lysostaphin treatments did not show any correlation with the sensitivity to methicillin and ampicillin. Lysostaphin exhibited good antibacterial activity, especially for MRSA-2, a clinical-isolated strain. Moreover, a highly drug resistant MRSA-3, which was insensitive to methicillin and ampicillin, remained sensitivity to lysostaphin, suggesting that evading the resistance mechanisms of existing antibiotics is a considerable advantage of lysostaphin.

**Fig. 1:**
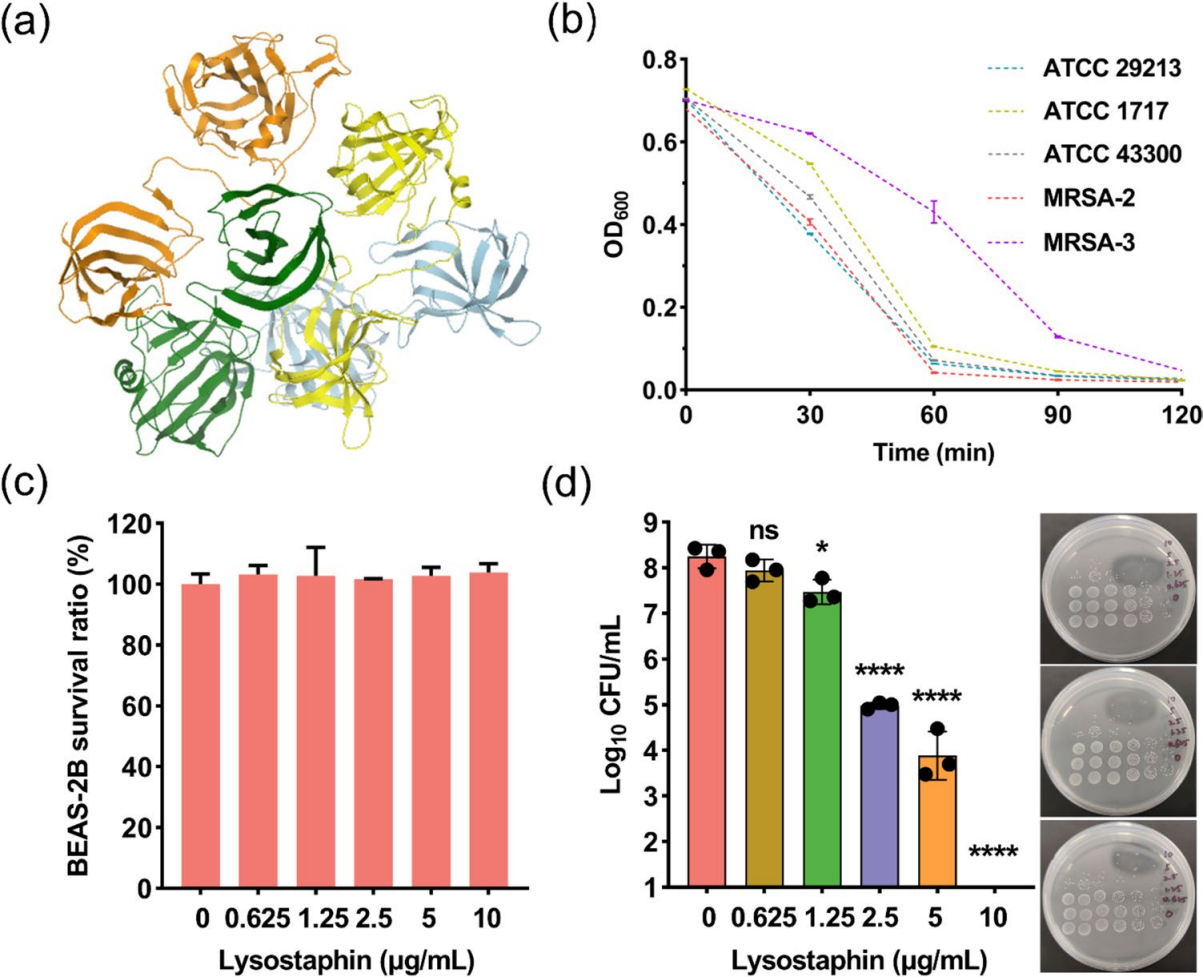
**a** The structure of lysostaphin (PDB code: 4LXC). **b** TRA of PBS-reconstituted lysostaphin against a panel of *S. aureus* strains. **c** Cytotoxicity of lysostaphin against BEAS-2B cells. **d** Antibacterial efficacy of lysostaphin in the BEAS-2B/MRSA-2 co-culture system. ns, no significant, *p < 0.05, ****p < 0.0001. For the Petri dishes, the vertical axis represents the concentration of lysostaphin and the horizontal axis represents bacterial colonies in a continuous tenfold dilution from high (lift) to low (right)

The structure of lysostaphin from *S. simulans* was shown in Fig. 1a. The activity of PBS-reconstituted lysostaphin was subsequently confirmed with TRA (Fig. 1b). It can be seen that in the presence of lysostaphin, OD_600_ values of all tested strains decreased from ~ 0.7 to less than 0.1 in 2 h, suggesting that lysostaphin could lead to rapid bacteriolysis.

*Staphylococcus aureus* has been found to be able to survive and propagate intracellularly, an important mechanism for them to escape the attack of antibacterial agents and leading to recurrent infections [23]. Thus, we investigated the anti-MRSA efficiency of lysostaphin in a co-culture system. Firstly, cytotoxicity of lysostaphin against human lung epithelial cells BEAS-2B was determined. As illustrated in Fig. 1c, lysostaphin showed an insignificant toxicity to this cell line at 10 μg/ml. Furthermore, in the BEAS-2B/MRSA-2 co-culture system (Fig. 1d), lysostaphin was able to reduce the CFU of MRSA-2 by nearly 1, 2, and 4 logs at the concentration of 1.25, 2.5, and 5 μg/ml, respectively. After treating with 10 μg/ml of lysostaphin for 2 h, no live MRSA-2 cells can be detected. These results suggested that lysostaphin could kill MRSA-2 effectively without affecting co-existed normal lung epithelial cells, further verifying its potential in treating MRSA-induced pulmonary bacterial infection.

### Short-term environmental tolerance of lysostaphin

**Fig. 2:**
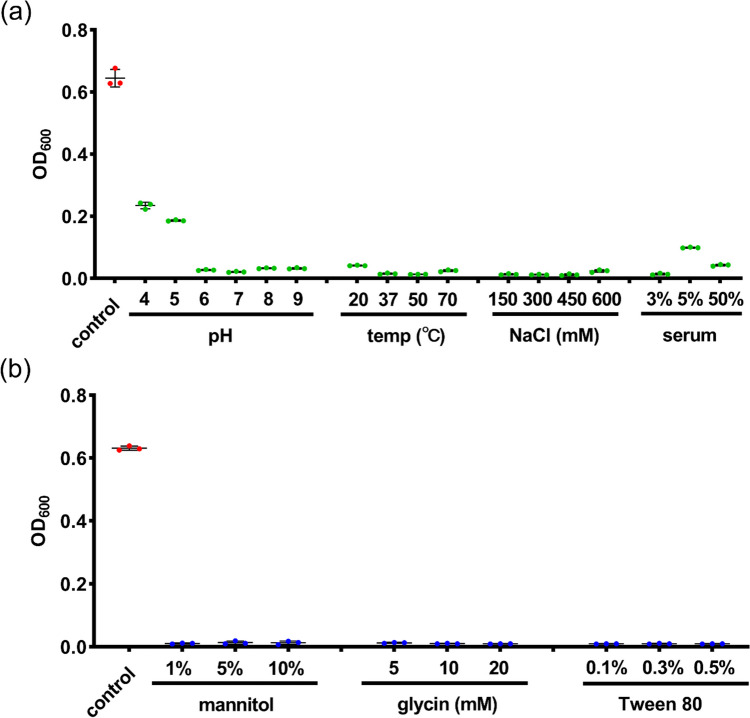
**a** OD_600_ values of lysostaphin-treated (4 μg/ml for 2 h) MRSA-2 at pH 4–9, temperature 20–70 °C, NaCl concentration 150–600 mM, and serum 3–50%. **b** OD_600_ values of lysostaphin-treated (4 μg/ml for 2 h) MRSA-2 at mannitol 1–10%, glycin 5–20 mM, and Tween 80 0.1 and 0.5%. MRSA-2 cells treated with PBS (pH 7.4, 25 °C) were set as control groups

Upon processing and application, lysostaphin may face different environmental stresses/challenges. Therefore, it is important to evaluate the stability of lysostaphin under different conditions. As shown in Fig. 2a (left panel), the enzyme remained fully active at alkaline and mild acidic conditions (pH 6–9), but the antibacterial activity started to decline as the pH decreased (pH 4–5). For healthy volunteers, the pH of the lower airway has been reported to be slightly alkaline (~ pH 7–8) and it changes in specific diseases [24, 25]. Patients with pneumonia were found to have a slightly lower pH (~ 6.6) [26–28]. According to our results, lysostaphin remains stable at these pH ranges, suggesting that it can retain its antibacterial activity at the lower airway to treat pneumonia. Next, we evaluated the stability of lysostaphin at different temperatures. Figure 2a (second panel) shows lysostaphin remained active after incubating at temperature ranged 20 to 70 °C, indicating their robustness against heat-generating processing within this range. The impact of ionic strength on the antibacterial activity of lysostaphin was also evaluated (Fig. 2a third panel). Negligible activity loss was found up to 600 mM NaCl, allowing a great extent of flexibility for its application and formulation for lung delivery. Serum intolerance is a major concern for most antibacterial enzymes, limiting them for local application. Lysostaphin appeared to have good tolerance with serum (Fig. 2a fourth panel), likely to retain activity in the lung environment in which the lung fluid consists of a complex mixture of proteins and other biological factors.

Excipients are important components of drugs and whether they will interfere with the active ingredients is an issue that must be investigated in the preliminary research. Herein, we evaluated whether excipients commonly employed in inhaled formulations, including mannitol, glycin, or Tween 80, would affect the bioactivity of lysostaphin against MRSA-2. Figure 2b revealed that lysostaphin performed favorable bacteriolysis even in the presence of high concentrations of mannitol (10%), glycin (20 mM), and Tween 80 (0.5%), suggesting that they can be used to formulate with lysostaphin without influencing its antibacterial effect. The ability of non-ionic surfactant, Tween 80, in preventing aggregation of protein in solution form have been well reported [29]. To minimize the usage of tween 80 without compromising the effect, PBS supplemented with 0.1% Tween 80 was selected to prepare the lysostaphin solutions. The aerosolization and storage stabilities of this liquid lysostaphin formulation were evaluated.

### Aerosolization of lysostaphin solution with a vibrating mesh nebulizer

**Fig. 3:**
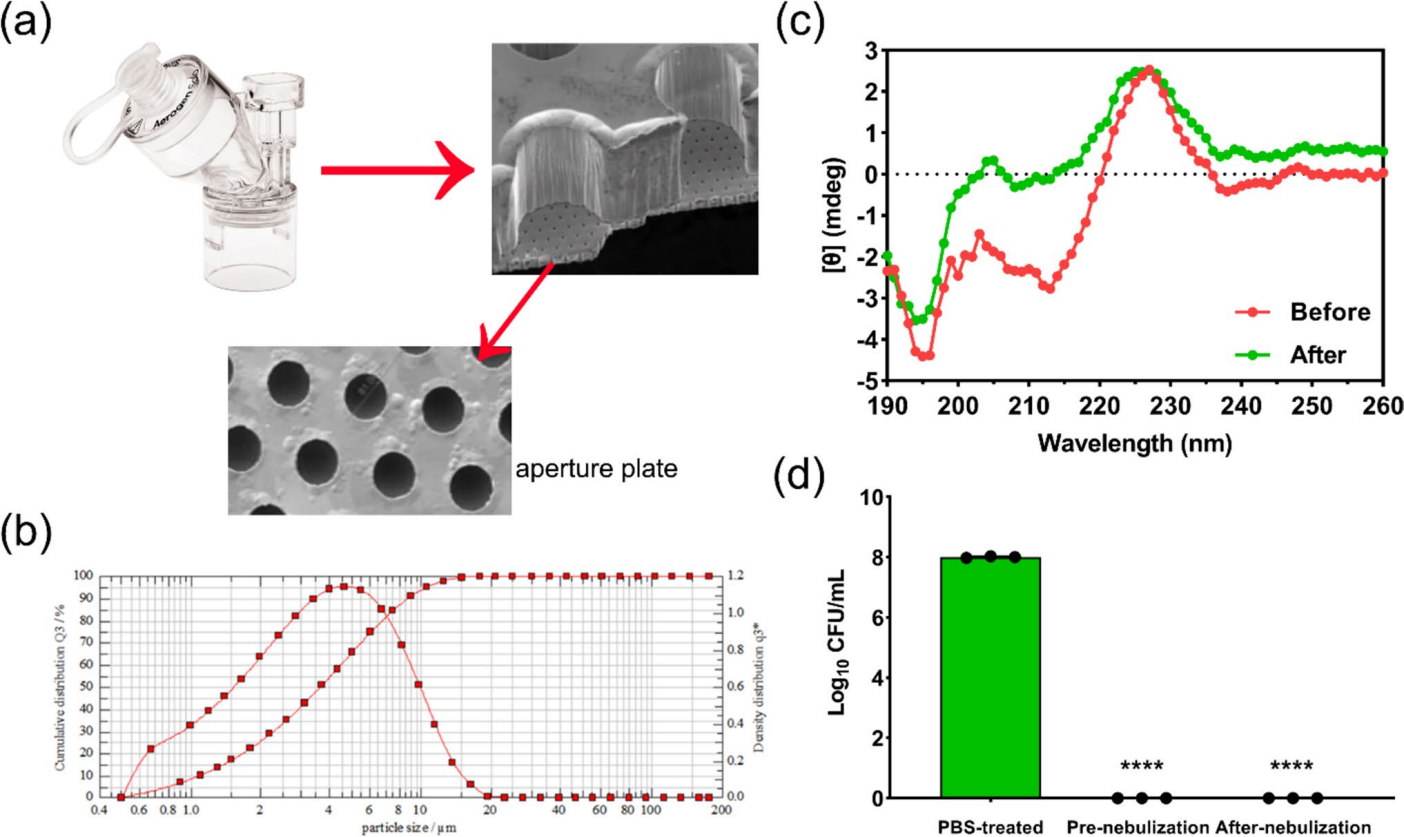
**a** Image and schematic of Aerogen^®^ Solo mesh nebulizer [30]. Reproduced with permission from Springer Nature. **b** Aerosol size distribution plot of the nebulized 1 mg/ml lysostaphin solution obtained by HELOS Sympatec. **c** CD spectra and **d** antibacterial efficiency of lysostaphin solutions before and after nebulization by VMN. ****p < 0.0001

A vibrating mesh nebulizer, Aerogen Solo^®^ (Fig. 3a), was used to nebulize the lysostaphin solutions at two distinct concentrations (0.3 and 1 mg/ml) into inhalable droplets. For mesh nebulization, the protein concentration is expected to change the tendency of protein aggregation and viscosity of the solution, hence the droplet size of the aerosols. Two protein concentrations were chosen for study to represent high (1 mg/ml) and low (0.3 mg/ml) concentration levels which may still be within the effective levels in lungs for bacterial killing. The Aerogen Solo^®^ nebulizer operates by the converse piezoelectric effect, wherein an applied voltage causes vibration of a piezoceramic ring element connected via a mesh washer to a 5-mm diameter mesh plate with 1000 dome-shaped apertures at ~ 128 kHz [30]. After powering up the nebulizer, the mesh plate vibrates up and down with ~ 1-μm vertical displacement, extruding the nearby liquid through the ~ 3-μm orifices in a micropump action to form aerosols. Aerosolized droplets intended for inhalation must have sizes ranging from 1 to 5 μm to achieve optimal deposition in the lungs. The geometrical sizes of lysostaphin aerosols generated by the Aerogen Solo^®^ nebulizer were determined by HELOS Sympatec (Fig. 3b). As shown in Table 2, the sizes of aerosol droplets were independent of the protein concentrations with more than half of the aerosols being inhalable (≤ 5 μm) [31]. Analysis of regional deposition prediction of aerosols showed lung depositions of ~ 43%.

**Table 2:** Size of aerosols generated by Aerogen Solo^®^ and Respimat^®^ SMI

Inhaler device	D_50_ (μm)	Span	Total deposition fraction (%)	Estimated lung deposition (%)
Aerogen Solo^®^ nebulizer-0.3 mg/ml	3.56 ± 0.07	2.09 ± 0.01	61.7 ± 0.6	43.0 ± 0.0
Aerogen Solo^®^ nebulizer-1 mg/ml	3.65 ± 0.07	2.10 ± 0.01	62.4 ± 0.6	43.1 ± 0.1
Respimat^®^ SMI	4.28 ± 0.18	1.67 ± 0.20	68.3 ± 1.3	48.9 ± 3.02

Protein and biological molecules are prone to loss their activity during the nebulization process, in which protein denaturation and aggregation may occur because of the generated shearing stresses [32]. Thus, the suitability of Aerogen Solo^®^ in delivery lysostaphin to the lungs was evaluated in the present study. To assess if the nebulization with the Aerogen^®^ Solo mesh nebulizer affects the structural stability of lysostaphin, we first analysed the secondary structure of the non-nebulized and nebulized proteins using CD spectroscopy, a widely used method to determine conformational changes of proteins [33]. As can be seen in Fig. 3c, CD spectra of lysostaphin maintained similar tendency of curve before and after nebulization, suggesting retention of the secondary structure. Figure 3d shows that the collected nebulized lysostaphin samples exhibited negligible antibacterial activity loss compared with the non-nebulized solution. Comparatively, Sweeney et al. [30] evaluated the utility of two VMNs developed by Aerogen, Solo^®^, and photo-defined aperture plate (PDAP^®^), in aerosolizing interferon gamma (IFN-γ) and confirmed negligible protein activity loss post-nebulization. Differently, Li et al. [34] found that both the Aerogen Solo^®^ and PARI VMNs failed to retain the activity of aerosolized proteins (IFN-α2, RBD-62, and BSA), with higher protein concentration showed better active protein recovery. They further showed that the addition of gelatin (0.5 and 2 mg/ml) could significantly stabilize the proteins upon nebulization to enhance the fractions of active proteins. These studies suggested that the activity loss of aerosolized proteins by mesh-vibrating nebulizers depended largely on the types of proteins and stabilizers used [35]. Taken together with the aerodynamic size distribution and the preservation of post-nebulization protein activity, the Aerogen^®^ Solo vibrating mesh nebulizer is considered a suitable inhaler device of in delivering this class of antibacterial into the lungs.

### Aerosolization of lysostaphin solution with a SMI

**Fig. 4:**
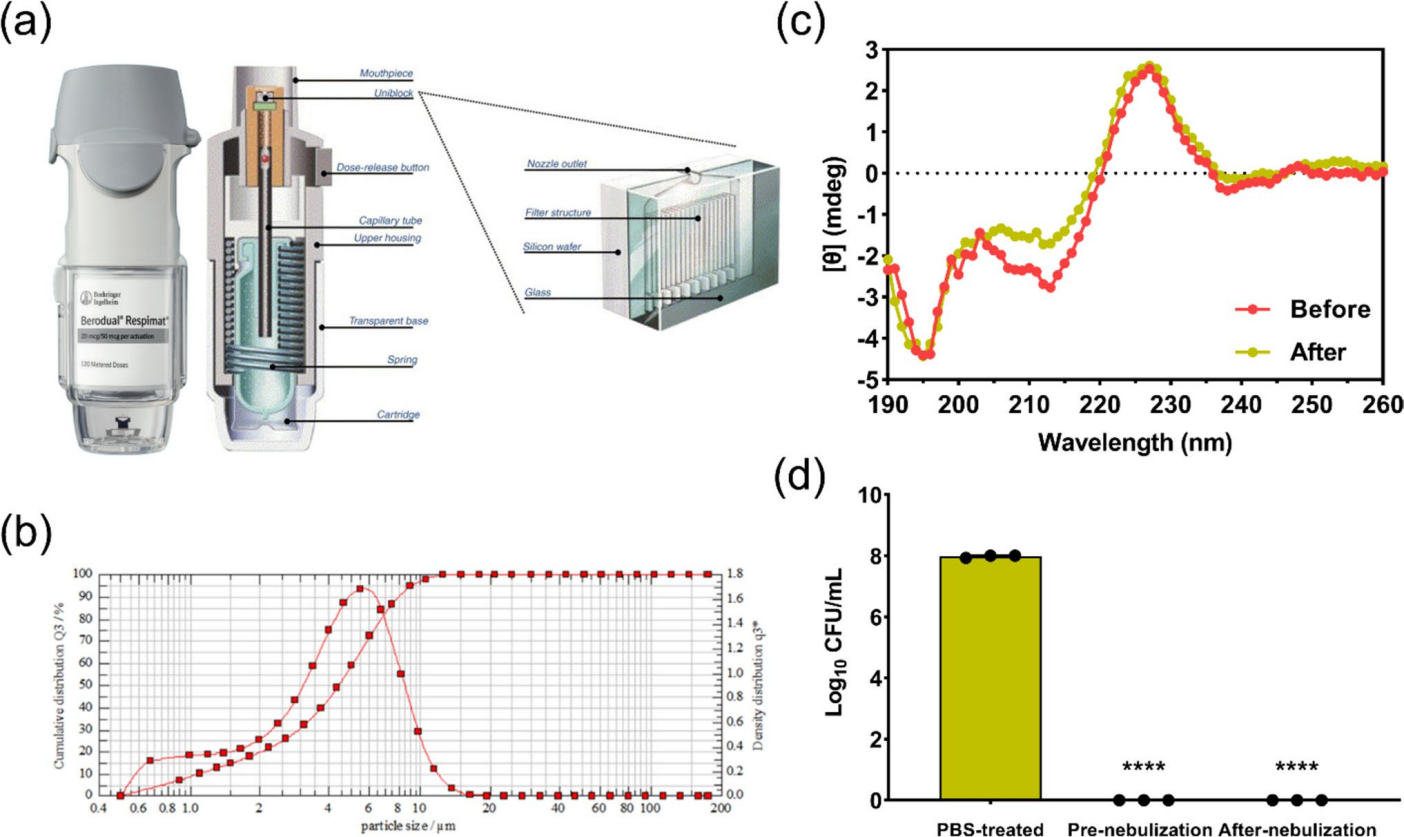
**a** Image and schematic of Respimat^®^ SMI [38]. Reproduced with permission from Elsevier. **b** Aerosol size distribution plot of the aerosolized 1 mg/ml lysostaphin solution obtained by HELOS Sympatec. **c** CD spectra and **d** antibacterial efficiency of lysostaphin solutions before and after nebulization by the SMI. ****p < 0.

Respimat^®^ SMI developed by Boehringer Ingelheim is a novel, multidose, propellant-free, hand-held, liquid inhaler required no battery. Figure 4a shows the relevant components of this device. Lysostaphin stock solution (1 mg/ml) were refilled into the drug cartridge that consists of an aluminum cylinder enclosing a double-walled polypropylene collapsible bag, which contracts as the solution is withdrawn for actuation [36]. When the base of a SMI is twisted 180°, a helical cam gear compresses a spring and lowers the capillary tube to transfer a pre-defined amount of drug solution (10–15 μl) into the dosing chamber across a one-way valve [36]. When the dose-release button is pressed, the mechanical energy from the compressed spring causes the capillary tube and one-way valve to move towards the nozzle system, named as “uniblock.” As a result, the drug solution in the dosing chamber is forced to flow through the microchannels within the uniblock, producing two fine jets at the outlet that collide at an optimized angle to form slowly moving aerosols with sizes suitable for inhalation (Fig. 4b and Table 2). Upon the aerosol generation process, pressure and shear are the major stresses acting on the proteins in SMI. While the suitability of SMI for the delivery of protein pharmaceuticals have not been explored before, a previous study evaluated the feasibility of using SMI to aerosolize D29 phage (95% of its components are proteins) and reported it caused no harm to the phage [37]. As shown in Fig. 4c, d, the pressure and shear in SMI were gentle enough to lysostaphin and did not cause significant change of its secondary structure, hence negligible activity loss in killing bacteria. Analysis of regional deposition prediction of aerosols delivered by the SMI showed lung depositions of ~ 48%, slightly higher than the mesh nebulizer. In general, according to the size of aerosolized droplets, estimated lung deposition ratio and antibacterial efficiency after nebulization, these two devices are both great lysostaphin inhaler with no significant difference.

### Storage stability of lysostaphin solution

**Fig. 5:**
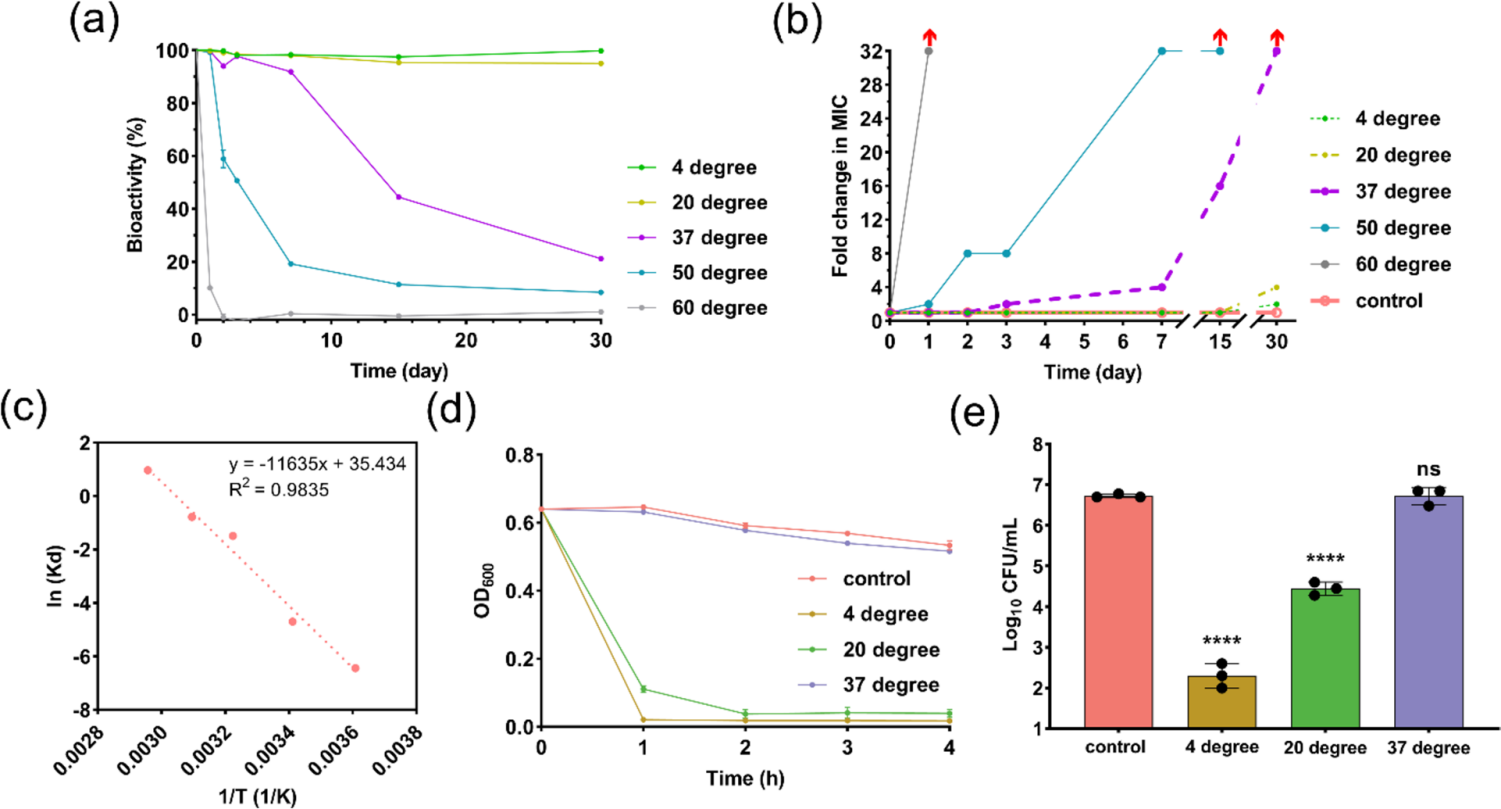
**a** Ratios of bioactivity retention of lysostaphin against MRSA-2 in varied storage temperatures. MRSA-2 treated with freshly prepared lysostaphin and PBS were defined as control groups (100% and 0%, respectively). **b** Fold change in MIC against MRSA-2 of lysostaphin stored at varied temperatures. MRSA-2 treated with freshly prepared lysostaphin was set as the control group. The red arrow presents beyond the detection limit (> 32-fold). **c** Arrhenius plot for shelf-life prediction. **d** TRA and **e** live colony counts of MRSA-2 treated with lysostaphin which stored at 4 °C, 20 °C, and 37 °C for 12 months. ns, no significant, ****p < 0.0001

Currently, lysostaphin is commercially available as lyophilized powder recommended to store at − 20 °C with a product shelf-life of 3 years. According to the U.S. FDA guideline [39], pharmaceutical products intended for storage in a freezer (− 20 ± 5 °C) should have a shelf-life of at least 12 months. This suggests lysostaphin can be formulated into powder form to meet this storage stability criteria and reconstituted for nebulization. However, the requirement of cold chain storage would increase the cost of storage and transportation. In addition, the formulation of lysostaphin for SMI application must be in the liquid form. We, therefore, evaluated the real-time storage stability of lysostaphin reconstituted in PBS + 0.1% Tween 80 at different temperatures. As depicted in Fig. 5a, negligible bioactivity loss was detected when lysostaphin solutions were stored at 4 °C and 20 °C for 30 days. However, if lysostaphin solution was stored at 37 °C, significant bioactivity decreases (> 50%) happened at day 15. As expected, the degradation rate of lysostaphin further increased at higher storage temperature that the bioactivities were dramatically reduced to < 20% in 7 days at 50 °C and in merely 1 day when stored at 60°C. The fold change in MIC shown in Fig. 5b was in line with the bioactivity loss data, collectively indicated that storage at 37 °C or above would result in noticeable inactivation of lysostaphin in 15 days. Heat treatment may inactivate protein in rather complex processes. Some enzymes have been reported to follow simple first-order kinetic model [40, 41]. Therefore, the thermal inactivation data of lysostaphin were analyzed kinetically together with the Arrhenius equation to see if lysostaphin followed simple first-order reaction. Table 3 shows the obtained thermodynamic parameters for the lysostaphin solution stored at different temperatures. The correlation coefficients (*R*^2^) of straight lines in Arrhenius plot (Fig. 5c) was 0.9835. The determined inactivation energy was 11.6 kJ/mol with an estimated half-life of 591.5 days for sample storing at 4 °C.

**Table 3:** Calculated thermodynamic parameters of lysostaphin solution at the tested temperature

Thermodynamic parameters	Temperature (°C)				
	4	20	37	50	60
${k}_{d}$ (/s)	0.0026	0.0091	0.225	0.4598	2.6492
${E}_{a}$ (kJ/mol)	11.6				
${t}_{shelf-life}$ (days)	591.5	57.7	6.3	1.4	0.3

Next, we validated the storage stability of lysostaphin reconstituted in PBS + 0.1% Tween 80 for a longer storage time (12 months). Figure 5d shows that this anti-staphylococcal enzyme exhibited good storage stability in the liquid state at 4 °C that minimum activity loss was noted after 12-month storage. Figure 5e further confirms the excellent storage stability at low temperatures (4 °C). Compared to the control group, the number of live bacterial colony decreased more than 4 logs, which meant that > 99% MRSA-2 cells were killed. As a comparison, lysostaphin stored at 20 °C for 12 months could still lead to approximately 2 logs reduction. These data support our assumption that for lysostaphin, storage in the liquid state at 4 °C is practicable.

## Conclusion

Pulmonary delivery of lysostaphin present a novel treatment strategy to manage MRSA-associated pneumonia. Based on the collected data, two inhalation devices, Aerogen^®^ Solo mesh nebulizer and Respimat^®^ SMI, were found to be suitable for the delivery lysostaphin into lungs and caused little impacts on its secondary structure hence its antibacterial activity. The choice of an appropriate inhaler device for lysostaphin delivery to lungs would then depend on the dose required. If only a small dose of lysostaphin (< 100 μg) is required, soft mist inhaler will be a favorable choice due to its portability and convenience. On the other hand, if a high dose (> 1 mg) lysostaphin is required for treatment, the mesh nebulizer will be a more suitable option. Accelerated stability test was adopted to estimate the shelf-life of lysostaphin solution stabilized by PBS and 0.1% Tween 80. The lysostaphin solution could be stable for at least 12 months.

## Data Availability

The datasets generated during and/or analyzed during the current study are available from the corresponding author on reasonable request.

## References

[1] WHO. The top 10 causes of death in 2019. 2019. Available at https://www.who.int/news-room/fact-sheets/detail/the-top-10-causes-of-death. Accessed 11 Jan 2023.

[2] Falcone M, Russo A, Giannella M, Cangemi R, Scarpellini MG, Bertazzoni G, Alarcón JM, Taliani G, Palange P, Farcomeni A, Vestri A, Bouza E, Violi F, Venditti M. Individualizing risk of multidrug-resistant pathogens in community-onset pneumonia. PLoS ONE. 2015;10:e0119528. 10.1371/journal.pone.0119528. 10.1371/journal.pone.0119528 PMC4393134 25860142

[3] Rubinstein E, Kollef MH, Nathwani D. Pneumonia caused by methicillin-resistant Staphylococcus aureus. Clin Infect Dis. 2008;46(Suppl 5):S378–85. 10.1086/533594. 10.1086/533594 18462093

[4] Jung WJ, Kang YA, Park MS, Park SC, Leem AY, Kim EY, Chung KS, Kim YS, Kim SK, Chang J, Jung JY. Prediction of methicillin-resistant Staphylococcus aureus in patients with non-nosocomial pneumonia. BMC Infect Dis. 2013;13:370. 10.1186/1471-2334-13-370. 23937553 10.1186/1471-2334-13-370 PMC3751064

[5] Sakamoto Y, Yamauchi Y, Jo T, Michihata N, Hasegawa W, Takeshima H, Matsui H, Fushimi K, Yasunaga H, Nagase T. In-hospital mortality associated with community-acquired pneumonia due to methicillin-resistant Staphylococcus aureus: a matched-pair cohort study. BMC Pulm Med. 2021;21:345. 10.1186/s12890-021-01713-1. 34732194 10.1186/s12890-021-01713-1 PMC8564271

[6] Chung DR, Huh K. Novel pandemic influenza A (H1N1) and community-associated methicillin-resistant Staphylococcus aureus pneumonia. Expert Rev Anti Infect Ther. 2015;13:197–207. 10.1586/14787210.2015.999668. 25578884 10.1586/14787210.2015.999668

[7] Roberts JC, Gulino SP, Peak KK, Luna VA, Sanderson R. Fatal necrotizing pneumonia due to a Panton-Valentine leukocidin positive community-associated methicillin-sensitive Staphylococcus aureus and Influenza co-infection: a case report. Ann Clin Microbiol Antimicrob. 2008;7:5. 10.1186/1476-0711-7-5. 18284686 10.1186/1476-0711-7-5 PMC2276235

[8] David MZ, Daum RS. Community-associated methicillin-resistant Staphylococcus aureus: epidemiology and clinical consequences of an emerging epidemic. Clin Microbiol Rev. 2010;23:616–87. 10.1128/cmr.00081-09. 20610826 10.1128/cmr.00081-09 PMC2901661

[9] Mediavilla JR, Chen L, Mathema B, Kreiswirth BN. Global epidemiology of community-associated methicillin resistant Staphylococcus aureus (CA-MRSA). Curr Opin Microbiol. 2012;15:588–95. 10.1016/j.mib.2012.08.003. 10.1016/j.mib.2012.08.003 23044073

[10] Mandell LA, Wunderink R. Methicillin-resistant staphylococcus aureus and community-acquired pneumonia: an evolving relationship. Clin Infect Dis. 2012;54:1134–6. 10.1093/cid/cis045. 22438344 10.1093/cid/cis045 PMC3309890

[11] McGuinness WA, Malachowa N, DeLeo FR. Vancomycin resistance in Staphylococcus aureus. Yale J Biol Med. 2017;90:269–81. 28656013 PMC5482303

[12] Shariati A, Dadashi M, Moghadam MT, van Belkum A, Yaslianifard S, Darban-Sarokhalil D. Global prevalence and distribution of vancomycin resistant, vancomycin intermediate and heterogeneously vancomycin intermediate Staphylococcus aureus clinical isolates: a systematic review and meta-analysis. Sci Rep. 2020;10:12689. 10.1038/s41598-020-69058-z. 32728110 10.1038/s41598-020-69058-z PMC7391782

[13] Bastos MD, Coutinho BG, Coelho ML. Lysostaphin: a staphylococcal bacteriolysin with potential clinical applications. Pharmaceuticals. 2010;3:1139–61. 10.3390/ph3041139. 27713293 10.3390/ph3041139 PMC4034026

[14] Wu JA, Kusuma C, Mond JJ, Kokai-Kun JF. Lysostaphin disrupts Staphylococcus aureus and Staphylococcus epidermidis biofilms on artificial surfaces. Antimicrob Agents Chemother. 2003;47:3407–14. 10.1128/aac.47.11.3407-3414.2003. 14576095 10.1128/aac.47.11.3407-3414.2003 PMC253758

[15] Kokai-Kun JF, Chanturiya T, Mond JJ. Lysostaphin as a treatment for systemic Staphylococcus aureus infection in a mouse model. J Antimicrob Chemother. 2007;60:1051–9. 10.1093/jac/dkm347. 17848374 10.1093/jac/dkm347

[16] Jayakumar J, Kumar VA, Biswas L, Biswas R. Therapeutic applications of lysostaphin against Staphylococcus aureus. J Appl Microbiol. 2021;131:1072–82. 10.1111/jam.14985. 33382154 10.1111/jam.14985

[17] Johnson CT, Sok MCP, Martin KE, Kalelkar PP, Caplin JD, Botchwey EA, García AJ. Lysostaphin and BMP-2 co-delivery reduces S. aureus infection and regenerates critical-sized segmental bone defects. Sci Adv. 2019;5:eaaw1228. 10.1126/sciadv.aaw1228. 10.1126/sciadv.aaw1228 PMC6524983 31114804

[18] Bastos MD, Coutinho BG, Coelho ML. Lysostaphin: a staphylococcal bacteriolysin with potential clinical applications. Pharmaceuticals. 2010;3(4):1139–61. 10.3390/ph3041139. 27713293 10.3390/ph3041139 PMC4034026

[19] Bodier-Montagutelli E, Mayor A, Vecellio L, Respaud R, Heuzé-Vourc’h N. Designing inhaled protein therapeutics for topical lung delivery: what are the next steps? Expert Opin Drug Deliv. 2018;15:729–36. 10.1080/17425247.2018.1503251. 10.1080/17425247.2018.1503251 30025210

[20] Marqus S, Lee L, Istivan T, Kyung Chang RY, Dekiwadia C, Chan HK, Yeo LY. High frequency acoustic nebulization for pulmonary delivery of antibiotic alternatives against Staphylococcus aureus. Eur J Pharm Biopharm. 2020;151:181–8. 10.1016/j.ejpb.2020.04.003. 32315699 10.1016/j.ejpb.2020.04.003

[21] Finlay WH, Martin AR. Recent advances in predictive understanding of respiratory tract deposition. J Aerosol Med Pulm Drug Deliv. 2008;21:189–206. 10.1089/jamp.2007.0645. 18518795 10.1089/jamp.2007.0645

[22] Shen W, Yang N, Teng D, Hao Y, Ma X, Mao R, Wang J. Design and high expression of non-glycosylated lysostaphins in Pichia pastoris and their pharmacodynamic study. Front Microbiol. 2021;12:637662. 10.3389/fmicb.2021.637662. 10.3389/fmicb.2021.637662 PMC8012855 33815324

[23] Fraunholz M, Sinha B. Intracellular Staphylococcus aureus: live-in and let die. Front Cell Infect Microbiol. 2012;2:43. 10.3389/fcimb.2012.00043. 22919634 10.3389/fcimb.2012.00043 PMC3417557

[24] Vaughan J, Ngamtrakulpanit L, Pajewski TN, Turner R, Nguyen TA, Smith A, Urban P, Hom S, Gaston B, Hunt J. Exhaled breath condensate pH is a robust and reproducible assay of airway acidity. Eur Respir J. 2003;22:889–94. 10.1183/09031936.03.00038803. 14680074 10.1183/09031936.03.00038803

[25] Zajac M, Dreano E, Edwards A, Planelles G, Sermet-Gaudelus I. Airway surface liquid pH regulation in airway epithelium current understandings and gaps in knowledge. Int J Mol Sci. 2021;22:3384. 10.3390/ijms22073384. 33806154 10.3390/ijms22073384 PMC8037888

[26] Abou Alaiwa MH, Reznikov LR, Gansemer ND, Sheets KA, Horswill AR, Stoltz DA, Zabner J, Welsh MJ. pH modulates the activity and synergism of the airway surface liquid antimicrobials β-defensin-3 and LL-37. Proc Natl Acad Sci USA. 2014;111:18703–8. 10.1073/pnas.1422091112. 25512526 10.1073/pnas.1422091112 PMC4284593

[27] Bodem CR, Lampton LM, Miller DP, Tarka EF, Everett ED. Endobronchial pH. Relevance of aminoglycoside activity in gram-negative bacillary pneumonia. Am Rev Respir Dis. 1983;127: 39–41. 10.1164/arrd.1983.127.1.39. 10.1164/arrd.1983.127.1.39 6849547

[28] Karnad DR, Mhaisekar DG, Moralwar KV. Respiratory mucus pH in tracheostomized intensive care unit patients: effects of colonization and pneumonia. Crit Care Med. 1990;18:699–701. 10.1097/00003246-199007000-00003. 2364709 10.1097/00003246-199007000-00003

[29] Agarkhed M, O’Dell C, Hsieh MC, Zhang J, Goldstein J, Srivastava A. Effect of polysorbate 80 concentration on thermal and photostability of a monoclonal antibody. AAPS PharmSciTech. 2013;14:1–9. 10.1208/s12249-012-9878-0. 23152309 10.1208/s12249-012-9878-0 PMC3581664

[30] Sweeney L, McCloskey AP, Higgins G, Ramsey JM, Cryan SA, MacLoughlin R. Effective nebulization of interferon-γ using a novel vibrating mesh. Respir Res. 2019;20:66. 10.1186/s12931-019-1030-1. 30943978 10.1186/s12931-019-1030-1 PMC6448243

[31] Morawska L, Johnson GR, Ristovski ZD, Hargreaves M, Mengersen K, Corbett S, Chao CYH, Li Y, Katoshevski D. Size distribution and sites of origin of droplets expelled from the human respiratory tract during expiratory activities. J Aerosol Sci. 2009;40(3):256–69. 10.1016/j.jaerosci.2008.11.002. 10.1016/j.jaerosci.2008.11.002 PMC7126899 32287373

[32] Shoyele SA, Slowey A. Prospects of formulating proteins/peptides as aerosols for pulmonary drug delivery. Int J Pharm. 2006;314:1–8. 10.1016/j.ijpharm.2006.02.014. 16563674 10.1016/j.ijpharm.2006.02.014

[33] Greenfield NJ. Using circular dichroism spectra to estimate protein secondary structure. Nat Protoc. 2006;1:2876–90. 10.1038/nprot.2006.202. 17406547 10.1038/nprot.2006.202 PMC2728378

[34] Li C, Marton I, Harari D, Shemesh M, Kalchenko V, Pardo M, Schreiber G, Rudich Y. Gelatin stabilizes nebulized proteins in pulmonary drug delivery against COVID-19. ACS Biomater Sci Eng. 2022;8:2553–63. 10.1021/acsbiomaterials.2c00419. 35608934 10.1021/acsbiomaterials.2c00419 PMC9159517

[35] Respaud R, Marchand D, Parent C, Pelat T, Thullier P, Tournamille JF, Viaud-Massuard MC, Diot P, Si-Tahar M, Vecellio L, Heuzé-Vourc’h N. Effect of formulation on the stability and aerosol performance of a nebulized antibody. MAbs. 2014;6:1347–55. 10.4161/mabs.29938. 10.4161/mabs.29938 PMC4623101 25517319

[36] Anderson P. Use of respimat soft mist inhaler in COPD patients. Int J Chron Obstruct Pulmon Dis. 2006;1:251–9. 10.2147/copd.2006.1.3.251. 18046862 10.2147/copd.2006.1.3.251 PMC2707154

[37] Carrigy NB, Chang RY, Leung SSY, Harrison M, Petrova Z, Pope WH, Hatfull GF, Britton WJ, Chan HK, Sauvageau D, Finlay WH, Vehring R. Anti-tuberculosis bacteriophage D29 delivery with a vibrating mesh nebulizer, jet nebulizer, and soft mist inhaler. Pharm Res. 2017;34:2084–96. 10.1007/s11095-017-2213-4. 28646325 10.1007/s11095-017-2213-4

[38] Dalby R, Spallek M, Voshaar T. A review of the development of Respimat Soft Mist Inhaler. Int J Pharm. 2004;283:1–9. 10.1016/j.ijpharm.2004.06.018. 15363496 10.1016/j.ijpharm.2004.06.018

[39] USFDA. Q1A(R2) Stability testing of new drug substances and products. 2003.

[40] Van Eylen D, Oey I, Hendrickx M, Van Loey A. Kinetics of the stability of broccoli (Brassica oleracea Cv. Italica) myrosinase and isothiocyanates in broccoli juice during pressure/temperature treatments. J Agric Food Chem. 2007;55:2163–70. 10.1021/jf062630b. 10.1021/jf062630b 17305356

[41] Ardila-Leal LD, Monterey-Gutiérrez PA, Poutou-Piñales RA, Quevedo-Hidalgo BE, Galindo JF, Pedroza-Rodríguez AM. Recombinant laccase rPOXA 1B real-time, accelerated and molecular dynamics stability study. BMC Biotechnol. 2021;21:37. 10.1186/s12896-021-00698-3. 34088291 10.1186/s12896-021-00698-3 PMC8178886

